# Dementia diagnosis and care across English memory services: A national survey

**DOI:** 10.1002/alz70860_099667

**Published:** 2025-12-23

**Authors:** Oliver Kelsey, Harriet Demnitz‐King, Charlotte kenten, Hannah Chapman, Malvika Muralidhar, Sedigheh Zabihi, Ellen Camboe, Elenyd M Whitfield, Annie Jones, Emma Williams, Susan Williams, Charles R Marshall, Jonathan M Schott, Ruth Dobson, Sube Banerjee, Claudia Cooper

**Affiliations:** ^1^ Queen Mary University of London, London, United Kingdom; ^2^ University of Liverpool, Liverpool, Liverpool, United Kingdom; ^3^ Queen Mary University of London, London, Greater London, United Kingdom; ^4^ University College London, London, England, United Kingdom; ^5^ University of Nottingham, Nottingham, United Kingdom

## Abstract

**Background:**

In England, most dementia diagnostic assessment and post‐diagnostic care are provided by NHS memory services, which offer free care at the point of delivery and serve >95% of the population. Unlike in many other countries, most patients in these services are seen by old age psychiatrists.

**Method:**

We co‐designed a survey about referral, diagnostic, and post‐diagnostic practices, and perceived readiness for new treatments. We invited all English memory services to complete it. We compared acute and community‐provider services. We compared service referral rates and staffing levels with catchment area and dementia diagnosis rate data, between regions; and explored whether service characteristics (provider type, rurality, NHS region, referral rates, staffing mix and accreditation) predicted diagnosis rates and psychological therapy provision.

**Result:**

139/188 (73.9%) memory services completed the survey, 130 (93.5%) provided by mental health/community trusts and 9 (6.6%) by acute trusts. Most (98%) of national referrals are to mental health/community‐based services, who report high annual referral rates (median 100.8, 56.7‐132.8/Full Time Equivalent [FTE]), doctors comprising 0.14 (0.09‐0.19) of FTE staff; and seeing 30% (20‐50%) of clients at home. Acute trust‐based services report fewer referrals (45.8, 21.1‐99.5) and more doctors (0.33, 0.23‐0.43) per FTE and saw few patients at home (0%, 0‐15%). Acute services felt more ready to prescribe dementia Disease Modifying Treatments than community services (8(88.9%) vs 50 (41.7%)) but were less likely to offer post‐diagnostic psychological therapy routinely (5(55%) vs 100(72.5%)). Preliminary analysis revealed that some NHS regions (*B* = 0.68, SE = 0.31, *p* = .031) and greater rurality (*B* = 1.84, SE=0.38, *p* = < .001) were associated with lower diagnostic rates in a regression model including all measured service characteristics, indicating geographical inequalities in service provision. Data analysis is ongoing, and we will present all findings at the conference.

**Conclusion:**

Current provision is patchy. Those living in rural areas have less access to timely diagnosis.

